# A mystery-shopping study to test enforcement of minimum legal purchasing age in Lithuania in 2022

**DOI:** 10.1093/eurpub/ckad027

**Published:** 2023-02-25

**Authors:** Laura Miščikienė, Alexander Tran, Janina Petkevičienė, Jürgen Rehm, Justina Vaitkevičiūtė, Lukas Galkus, Shannon Lange, Mindaugas Štelemėkas

**Affiliations:** Health Research Institute, Faculty of Public Health, Lithuanian University of Health Sciences, Kaunas, Lithuania; Department of Health Management, Faculty of Public Health, Lithuanian University of Health Sciences, Kaunas, Lithuania; Institute for Mental Health Policy Research, Centre for Addiction and Mental Health, Toronto, Canada; Health Research Institute, Faculty of Public Health, Lithuanian University of Health Sciences, Kaunas, Lithuania; Department of Preventive Medicine, Faculty of Public Health, Lithuanian University of Health Sciences, Kaunas, Lithuania; Institute for Mental Health Policy Research, Centre for Addiction and Mental Health, Toronto, Canada; Campbell Family Mental Health Research Institute, Centre for Addiction and Mental Health, Toronto, Canada; Department of Psychiatry, University of Toronto, Toronto, Canada; Faculty of Medicine, Institute of Medical Science, University of Toronto, Toronto, Canada; Dalla Lana School of Public Health, University of Toronto, Toronto, Canada; Institute of Clinical Psychology and Psychotherapy & Center of Clinical Epidemiology and Longitudinal Studies (CELOS), Technische Universität Dresden, Dresden, Germany; Center for Interdisciplinary Addiction Research (ZIS), Department of Psychiatry and Psychotherapy, University Medical Center Hamburg-Eppendorf (UKE), Hamburg, Germany; Program on Substance Abuse, Public Health Agency of Catalonia, Program on Substance Abuse & designated WHO CC, Public Health Agency of Catalonia, Barcelona, Spain; Health Research Institute, Faculty of Public Health, Lithuanian University of Health Sciences, Kaunas, Lithuania; Health Research Institute, Faculty of Public Health, Lithuanian University of Health Sciences, Kaunas, Lithuania; Institute for Mental Health Policy Research, Centre for Addiction and Mental Health, Toronto, Canada; Campbell Family Mental Health Research Institute, Centre for Addiction and Mental Health, Toronto, Canada; Department of Psychiatry, University of Toronto, Toronto, Canada; Health Research Institute, Faculty of Public Health, Lithuanian University of Health Sciences, Kaunas, Lithuania; Department of Preventive Medicine, Faculty of Public Health, Lithuanian University of Health Sciences, Kaunas, Lithuania

## Abstract

**Background:**

According to the Lithuanian law to prevent the sale of alcohol to customers below the legal minimum purchasing age of 20 years, young adults below 25 years must be asked to show an age-verification document when purchasing alcohol. The aim of this study was to assess whether off-premise outlets comply with the law.

**Methods:**

In 2022, mystery-shopping study was carried out in three consecutive phases: (i) in a representative sample (*n* = 239) of off-premise alcohol outlets covering all Lithuanian district centres, (ii) after lifting the requirement to wear a mask and (iii) after warning the outlets that a mystery-shopping study was ongoing. Phases 2 and 3 were held in two cities. The mystery shopping involved attempts by young, but legally eligible customers to purchase alcohol. Across the three study phases, we compared compliance with the law by measuring overall success of purchase attempts and included situational characteristics (working day or weekend), time of day and number of customers in line as an additional predictor.

**Results:**

Out of 239 attempts to purchase alcohol from off-premise outlets in the main phase of the study, 107 (or 44.8%) were considered to be successful (visits in which staff were willing to sell alcohol). There was a significantly higher chance of success to purchase alcohol with no ID request if a mystery shopper was the only customer in a queue and on weekends.

**Conclusions:**

The results indicate an insufficient level of age-verification control in Lithuania, and that additional action is needed to increase compliance.

## Introduction

The period of 2016–18 in Lithuania was marked by the implementation of several significant alcohol control policies. Beginning in 2016, alcohol sales in petrol stations were fully banned, followed by a major increase in excise taxation starting in March 2017. In 2018, a ban on alcohol marketing came into effect (complete ban of TV, radio and digital advertisements with only a few exceptions like memorabilia), along with reduction in off-premise sale hours (from 8:00–22:00 during the week to 10:00–20:00 from Monday to Saturday and 10:00–15:00 on Sunday), and an increase in the minimum legal alcohol purchasing and drinking age, from 18 to 20 years old.^1^

In addition to the increased minimum legal alcohol purchasing age, the retailers were also obligated by the Law on Alcohol Control to verify a purchaser’s age if the person appeared to be younger than 25 years old. The increase in minimum legal drinking age, along with the rules associated with age verification, resulted into intense debates between policymakers and various interest groups (health policy advocates, the alcohol industry and retail sale lobbyists). In general, industry was lobbying for more liberal age regulation with respect to the purchasing of alcohol but the planned regulation was implemented as the strengthening of alcohol control policies was one of the electoral promises by the ruling political party at the time.^1^ The public surveys tend to show the support for the implemented alcohol control policies, especially for the increased minimum legal alcohol purchase age.^2^

Younger age of drinking onset may contribute to alcohol-related problems, and thus deciding on the minimum legal drinking (purchasing) age is critical,^3–5^ and additional regulations, such as age verification is a key to prevent alcohol purchases by minors. The previous study had suggested that there was a significant effect of the minimum legal drinking age on all-cause mortality rates in those 18–19 years old.^6^ Although having regulations in place is important, it also must be actively enforced. Currently, in Lithuania, the minimum legal drinking restrictions may be insufficiently accompanied by active enforcement strategies regarding age verification to comply with the law. This sets the background for a mystery-shopping study in Lithuania, a country with no systematic monitoring on its alcohol purchasing age enforcement practices. No similar studies were carried out in Lithuania.

Compliance with the minimum legal drinking age using the mystery-shopping method has been tested in several other European countries. In a study by Rossow et al.,^7^ in Finland and Norway, under-age-appearing 18-year-olds attempted to purchase alcohol in off-premise outlets. Outcomes were measured as to whether or not the buyers were asked to present an ID card and whether or not they succeeded in purchasing alcohol. The mystery shoppers were asked to present an ID card in slightly more than half the attempts, and they succeeded in purchasing alcohol in 48% of the cases.

Van Hoof et al.^8^ reported on measuring vendors’ compliance and possible changes in the level of compliance with the legal age limits on alcohol sales in the Netherlands in 2011 and 2013. When testing the rate of compliance in 2737 alcohol purchase attempts made in both on- and off-premise outlets as well as from alcohol home delivery services, they found that the ID request increased from 43.9% in 2011 to 54.1% in 2013.^8^ Later, Offermans et al.^9^ investigated whether raising the minimum legal age for the sale of alcohol had influenced compliance rates among Dutch alcohol vendors. According their results, alcohol sellers’ compliance rates increased for 15-year-old buyers from 46.5% to 55.7% and then to 73.9% 1 and 2 years after the policy implementation, respectively.^9^

Another study from Spain reported a possible association between the implementation of a multicomponent intervention to reduce adolescents’ alcohol use, and the sale of alcohol to minors. Adolescents conducted test purchases in 2018 (baseline; *n* = 73) prior to the intervention, and again in January 2020 (follow-up; *n* = 65). In the supermarkets with an intervention programme, asking for proof of age significantly increased, from 15.4% to 72.7%.^10^

The aim of this study was to evaluate the level of compliance with existing regulations on age verification in Lithuania, after the legal purchasing age was increased to 20 years of age, for purchasers who appear to be younger than 25 years of age. In addition, based on similar studies conducted in Europe and the results of a pilot study in Kaunas (Lithuania), we tested the following two hypotheses: (i) more than half of supermarkets and grocery stores would not comply with the law to ask the person purchasing the alcoholic beverage to provide an identity document when there a person was potentially under the age of 25. (ii) After an intervention notifying the stores about the existence of a secret shopper experiment, there would be a significant increase in the proportion of supermarkets and grocery stores, which would comply with the law requiring age verification with an identity document when there was a strong possibility that the shopper was under the age of 25.^11^

## Methods

### Mystery-shopping study protocol

Mystery shoppers. The mystery shopping involved attempts by young, but legally eligible (20–24 years old) customers to purchase alcohol, and to observe whether staff requested ID prior to completing the sale. Although the mystery shoppers were of legal age, they should appear to be young enough to trigger a request for ID. Underage mystery shoppers were not recruited to ensure that attempted purchases posed no legal/ethical conflicts.

Recruitment of mystery shoppers. We recruited University students of chosen age group. Eight female students participated in the research. The mean of the mystery shoppers age 21.13 (SD = 0.593). Training of the mystery shoppers was conducted on alcohol test purchase protocol and questionnaire (see Supplementary appendix 3 table S1). The actions of the mystery shoppers were trained according to the Mystery-shopping decision-making flowchart (see Supplementary appendix 2 figure S2).

Sample. Based on state alcohol licencing data (8046 licences issued for off-premise sales), we randomly sampled 239 off-premise retailers for the study. The sample included five of the biggest grocery-store chains in Lithuania. We chose central cities (Vilnius, Kaunas, Klaipėda, Šiauliai, Marijampolė, Panevėžys, Alytus, Utena, Telšiai and Tauragė) of all 10 counties in Lithuania and determined the number of attempts in each city based on the total number of stores in each city. Grocery stores were randomly selected from all operating stores.

Intervention. Visits were scheduled at various times of day and days of the week. With each visit, mystery shoppers tried to buy a randomly chosen brand of beer, or wine, as it was determined that those are the preferred drinks for women in the selected age range in Lithuania.^12^

When approaching the checkout, mystery shoppers counted the number of customers in the line before them, and, once the store personnel started serving them, the number of customers behind them. If the store personnel asked the shopper’s age, mystery shopper answered truthfully. If asked for an ID, the mystery shopper claimed not to have one with them at that time. If the store personnel refused to complete the transaction without an ID, mystery shopper did not insist further and left the store. If the store personnel were willing to complete the transaction without an ID, the mystery shopper stopped the purchase process. Visits in which the store personnel refused to sell alcohol without presentation of valid ID were coded as ‘fail’, while visits in which store personnel were willing to sell will be coded as ‘success’.

No real purchase of alcohol took place, but the unambiguous selling intentions were used as indications of real alcohol sales. Immediately after the store visit, the mystery shopper filled out a checklist (Supplementary appendix S2).

Self-service points were not included in this study to maintain the consistency across the cities and towns and across the supermarkets (the self-service option is more concentrated in bigger stores and the level of development of these services varies across the different grocery-store chains).

### Research phases

The mystery-shopper study consisted of three research phases (see Supplementary appendix 1 figure S1): (i) a pilot study was implemented to test the study protocol. The pilot study took place in grocery stores in Kaunas, involved eight secret shoppers, and resulted in 36 purchase attempts in December 2021 with the ID request rate of close to 64% of cases. To test our hypothesis that: more than half of supermarkets and grocery stores would not comply with age-verification laws when there is a doubt that a person is under the age of 25; we had a first phase visits, in February 2022 (*n* = 239). As research was conducted during Covid-19 pandemic, there was a requirement to wear a mask. In order to check if masks could have an effect on store personnel behaviour, the second phase was composed of additional visits to two cities (*n* = 73) as soon as the mask requirement was lifted in a county. Cities were chosen based on the results from the first phase (i.e. one major city with more successful attempts and one with higher unsuccessful purchase rate were identified). Additionally, Phase 3 took place in May 2022, to test the effect of an intervention notifying the communications department of each grocery-store chain about the mystery-shopper experiment. We expected that there would be a significant increase in a proportion of supermarkets and grocery stores, which complied with the law to present age verification when purchasing alcoholic beverages if there was doubt that a person was under the age of 25. We chose the same two cities (*n* = 73) from Phases 1 and 2 to have an exact match across the three phases. At this research phase, emails about the mystery-shopper visits to stores were sent prior to the store visits, to these departments (Supplementary appendix S4) and after a few days the stores were visited. The stores visited were the same in Phases 1 and 2; however, different mystery shoppers were used.

To confirm that the mystery shoppers were of ambiguous legal purchasing age (i.e. appeared to be under the age of 25), we tested inter-rater reliability, using intraclass correlation coefficient (ICC). Study involved five raters of 35–50 years old (the average age of the employees in the study was ∼40 as indicated by the mystery shopper). Raters estimated the ages of random pictures of the mystery shoppers’ faces, faces of similar ages and older or younger faces (*n* = 32). Raters estimated the ages of the faces using age categories (first group of people up to 20 years; second=20–24 years old; third=25 years and older). We found the level of reliability among raters for age estimation for the mystery shoppers’ faces to be high [ICC = 0.889 with a 95% confidence interval (95% CI) from 0.785 to 0.944]. These findings indicated that the mystery shoppers’ faces would reasonably be identified as young people by store personnel, and therefore should have resulted in a request for ID in grocery stores when purchasing alcoholic beverages.

### Data and statistical analysis

Statistical data analysis was performed using the statistical package IBM SPSS Statistics for Windows, Version 20.0 (2011 version, IBM Corp, Armonk, NY, USA) and Microsoft Excel 2019 (Microsoft, Redmond, WA, USA). A statistical significance level (*P*-values) of 0.05 was chosen to test the hypotheses. The qualitative variables were presented as percentages. Differences in qualitative variables were compared using Chi-squared (*χ*^2^) tests. The categorical variables were presented as percentages and compared using *χ*^2^ and *Z* tests with Bonferroni correction. The Clopper–Pearson interval was used to calculate binomial 95% CIs. ICC estimates and their 95% CIs were calculated based on average-measures, absolute-agreement, two-way mixed-effects model.

## Results

The main part (Phase 1) of the study took place between February and March of 2022, and was implemented in 10 county central cities with a total of 239 attempts to purchase alcohol beverages (beer or wine). In total, in 104 (43.5%) of the purchase attempts, the store personnel did not ask the mystery shoppers for their IDs (table 1). In five cases there were successful purchase attempts when ID was requested but not provided by the mystery shopper, and in two cases the sale was refused without the seller requesting ID. There were two successful attempts to buy alcohol when the age of mystery shopper was asked, and 105 successful attempts when the store personnel did not ask the age of the mystery shopper. A significant effect of ID requests and successful attempts of alcohol purchases was observed (*P* < 0.001), which resulted in 107 successful alcohol purchases (44.8% of all attempts) at the main phase (Phase 1) of the study (table 1).

**Table 1:** Distribution of successful and unsuccessful attempts to buy alcohol by shopping characteristics (the results of the main phase of the study)

	Successful attempt			Unsuccessful attempt			Total
	*N*	%	95% CI	*N*	%	95% CI	*n* (%)
Did store personnel ask for ID?							
Yes	5	3.7	1.2–8.4	130	96.3	91.6–98.8	135 (100)
No	102	98.1	93.2–99.8	2	1.9	0.2–6.8	104 (100)
Total	107	44.8	38.4–51.3	132	55.2	48.7–61.6	239 (100)
Chi-squared (*χ*^2^) test	*P* *<*0.001						
Did the store personnel ask the age of the mystery shopper?							
Yes	2	10.5	1.3–33.1	17	89.5	66.9–98.7	19 (100)
No	105	47.7	41.0–54.5	115	52.3	45.5–59.0	220 (100)
Chi-squared (*χ*^2^) test	*P* *=* 0.002						
Time of the day							
Until 17 h	84	45.2	37.9–52.6	102	54.8	47.4–62.1	186 (100)
17 h and later	23	43.4	29.8–57.7	30	56.6	42.3–70.2	53 (100)
Chi-squared (*χ*^2^) test	*P* *=* 0.820						
Weekday							
Workday	58	39.5	31.5–47.8	89	60.5	52.2–68.5	147 (100)
Weekend	49	53.3	42.6–63.7	43	46.7	36.3–57.4	92 (100)
Chi-squared (*χ*^2^) test	*P* *=* 0.037						
Customers in line behind mystery shopper							
0	45	54.9^a^	43.5–65.9	37	45.1^a^	34.1–56.5	82 (100)
1	27	40.3	28.5–53.0	40	59.7	47.0–71.5	67 (100)
2	13	29.5	16.8–45.2	31	70.5	54.8–83.2	44 (100)
3 and more	22	47.8	32.9–63.1	24	52.2	36.9–67.1	46 (100)
Chi-squared (*χ*^2^) test	*P* *=* 0.042						
Number of customers in line in front of mystery shopper							
0	15	46.9	29.1–65.3	17	53.1	34.7–70.9	32 (100)
1	33	50.0	37.4–62.6	33	50.0	37.4–62.6	66 (100)
2	22	39.3	26.5–53.2	34	60.7	46.8–73.5	56 (100)
3 and more	37	43.5	32.8–54.7	48	56.5	45.3–67.2	85 (100)
Chi-squared (*χ*^2^) test	*P* *=* 0.677						

a *Z*-test with Bonferroni correction, significant difference between zero customers in line behind mystery shopper and two customers in line behind mystery shopper (*P* *<* 0.05).

When comparing the other parameters of the purchases, we found that the success rate of purchasing attempts was significantly higher on the weekend (*P* = 0.037), but there were no differences found between times of purchase (see table 1).

Finally, table 1 also summarizes the impact of a waiting line to an overall successful purchase. We observed that the highest proportion of successful attempts was when there were no other customers in line behind the mystery shopper (54.9%; a similar proportion was found for situations where ID was not requested, see table 2). The number of customers in front of the mystery shopper when waiting in a queue did not have an impact on a successful purchase (see tables 1 and 2).

**Table 2:** Distribution of request to show ID by number of customers in waiting line (analyzing the results of the main phase of the study)

	Did store personnel asked for an ID?						Total
	No			Yes			
	*N*	%	95% CI	*N*	%	95% CI	*N* (%)
Number of customers in line behind mystery shopper							
0	45	54.9	43.5–65.9	37	45.1	34.1–56.5	82 (100)
1	24	35.8	24.5–48.5	43	64.2	51.5–75.5	67 (100)
2	14	31.8	18.6–47.6	30	68.2	52.4–81.4	44 (100)
3 and more	21	45.7	30.9–61.0	25	54.3	39.0–69.1	46 (100)
Chi-squared (*χ*^2^) test	*P* *=* 0.037						
Number of customers in line in front of mystery shopper							
0	16	50.0	31.9–68.1	16	50.0	31.9–68.1	32 (100)
1	29	43.9	31.7–56.7	37	56.1	43.3–68.3	66 (100)
2	22	39.3	26.5–53.2	34	60.7	46.8–73.5	56 (100)
3 and more	37	43.5	32.8–54.7	48	56.5	45.3–67.2	85 (100)
Chi-squared (*χ*^2^) test	*P* *=* 0.811						

Next, in Phase 2 of the study, we tested the impact of lifting the requirement to wear a mask in a grocery store, and in Phase 3 of the study, we tested what impact notification of the mystery-shopping experiment there might be on ID requests and the overall success rate of alcohol purchases. Table 3 summarizes the results. In short, there was no significant difference found between the three different phases of this study [i.e. buying success when comparing purchase attempts in the same two cities in all three phases did not differ significantly (*P* = 0.704)], nor were there any differences between data in each of the three phases (*P* = 0.863). Also, we found no differences between research phases and whether store personnel asked mystery shoppers for ID (*P* = 0.876, see table 4).

**Table 3:** Distribution of successful and unsuccessful attempts to buy alcohol by research phases (%)

	Successful attempt			Unsuccessful attempt			Total
	*N*	%	95% CI	*N*	%	95% CI	*n* (%)
Research phase							
Phase 1 (main phase, using data from Klaipėda and Kaunas only)^a^	30	41.1	29.7–53.2	43	58.9	46.8–70.3	73 (100)
Phase 2 (without masks, in Klaipėda and Kaunas)	35	47.9	36.1–60.0	38	52.1	40.0–63.9	73 (100)
Phase 3 (after notification intervention, in Klaipėda and Kaunas)	32	43.8	32.2–55.9	41	56.2	44.1–67.8	73 (100)

a Chi-squared (*χ*^2^) test; *P* *=* 0.704.

**Table 4:** Distribution of request to show an ID (%)

	Did store personnel asked for an ID?						Total
	No			Yes			
	*N*	%	95% CI	*N*	%	95% CI	*N* (%)
Research phase							
Phase 1 (main phase, using data from 10 cities/towns)	104	43.5	37.1–50.1	135	56.5	49.9–62.9	239 (100)
Phase 2 (without masks)	30	41.1	29.7–53.2	43	58.9	46.8–70.3	73 (100)
Phase 3 (after intervention)	31	42.5	31.0–54.6	42	57.5	45.4–69.0	73 (100)
Total	165	42.9	37.9–48.0	220	57.1	52.0–62.1	385 (100)
Chi-squared (*χ*^2^) test	*P* *=* 0.933						
Research phase (in Kaunas and Klaipėda only)							
Phase 1 (main phase, using data from Klaipėda and Kaunas only)	28	38.4	27.2–50.5	45	61.6	49.5–72.8	73 (100)
Phase 2 (without masks)	30	41.1	29.7–53.2	43	58.9	46.8–70.3	73 (100)
Phase 3 (after intervention)	31	42.5	31.0–54.6	42	57.5	45.4–69.0	73 (100)
Total	89	40.6	34.1–47.5	130	59.4	52.5–65.9	219 (100)
Chi-squared (*χ*^2^) test	*P* *=* 0.876						

## Discussion

In Phase 1 (main phase) of this study, with data collected in 10 towns and cities, we found that 44.8% of alcohol purchase attempts were successful. Thus, the final success rate was lower than we had hypothesized. However, nearly one out of two mystery shoppers could have successfully purchased alcohol, despite being within the age range for which ID should have been requested—a situation which would considerably undermine the effectiveness of the legislation. This creates implications for underage shoppers to purchase alcohol when ID is not requested as intended by the requirements of the law. However, this specific aspect cannot be easily tested in the current legal situation in Lithuania, as only dedicated institutions like police have the right to imitate a violation of the law, thus, we only implement a mystery-shopper experiment only by inviting the participants who are slightly above the legal age to purchase alcohol.

When looking to other European countries, compliance and enforcement of age-verification legislation is not an issue in Lithuania alone. In the Netherlands, compliance of age verification has very slowly increased over time. Schelleman-Offermans et al.^9^ found that the age-verification compliance (purchasing beer, mix, wine, or spirits) in the Netherlands after the minimum legal age was increased from 16 to 18 years old in 2014 was 46.5%, which increased to 74% 2 years later. In a recent study by Kamin et al. (2021),^13^ in Slovenia the rate of compliance (purchasing beer and a snack) was just over 11%; which improved after a compliance-enhancing intervention, to over 20%. In contrast to Europe, the results from the four states in the USA indicated that compliance with the ID check requirement may be above 80%.^14^

Thus, the international experience shows that the compliance may be improved with a compliance-enhancing intervention. We did not observe an impact from grocery-store chain notification in this study, meaning stronger interventions may be required, and this may be an area for future studies. The preliminary results of this study were disseminated to the national control institution, to grocery-store chain communication departments and to media outlets with the aim of informing the general Lithuanian public about the study results.^15^ This resulted in a number of national internet media messages, and may be a starting point for a more systematic monitoring initiative in a country. To our knowledge, there was only one attempt to do a compliance experiment in Lithuania prior to this one. In 2015, an underage customer in an unstructured experiment attempted to purchase beer in 17 petrol stations and was successful in 14 of the attempts.^16^ The experiment gained significant media attention due to a period of public debate about whether it was necessary to ban sales of alcohol in petrol stations in Lithuania (see Miščikienė et al.^1^ for more details).

In addition, we analyzed the impact of the waiting lines, and the biggest proportion of successful attempts was observed when there were no other customers in line behind the mystery shopper (54.9%). This result may be explained from a behavioural psychology perspective where store personnel tend to show more responsible behaviour (according to the legal requirements) when they are seen by a higher number of people.^17^ As well, these findings suggest that the pressure of a large customer line-up, and resulting pressure to work faster (i.e. skip the ID verification), may not play a significant role in these scenarios.

There are a number of limitations that need to be considered when interpreting the results. First, the majority of the purchase attempts were during the period when there was still a requirement for on-premise mask-wearing due to pandemic restrictions. However, we introduced a follow-up study to test the impact of masks, which indicated that there was no effect of mask-wearing on compliance. Second, there were only female mystery shoppers that were study volunteers, and thus future studies should attempt to recruit men and gender-diverse individuals as secret shoppers. Third, the mystery shoppers were instructed to purchase wine or beer only, and this may have provided a different compliance rate than with higher alcoholic-content beverages. Fourth, future studies should include specialized alcohol stores and self-service checkout spaces. Finally, the focus of this study was on purchases in urban centres (large cities and towns), and we mainly focussed on five major grocery-store chains. However, presumably, there would be similar rates in smaller alcohol outlets (where the success of the business is even more dependent on sales numbers). Despite the above limitations, we believe that this study was a rigorous test of the enforcement of age-verification legislation on alcohol purchase attempts and can inform future policy-making and enforcement decisions in Lithuania.

In summary, the study results indicate that there may be an insufficient level of compliance in Lithuania regarding the formal requirement to verify the age if a customer purchasing an alcoholic beverage appears to be younger than 25 years old. The current approach when legal requirement is not led by clear enforcing strategies by the state, may be not sufficient. Circulating simple message to grocery-store chains informing them about the upcoming mystery-shopper experiment did not result into any significant effect, which suggests a need for more comprehensive engagement with the stakeholders in the future.

## Supplementary Material

ckad027_Supplementary_Data

## Data Availability

The data underlying this article are available in the article and in its online Supplementary material. Key points The study showed that close to 45% of underage mystery shoppers were considered to be successful in purchasing alcohol when ID was not requested. Informing store chains about the upcoming mystery-shopper experiment did not result into any significant change in results of the compliance. The study has indicated that there is a need for constant monitoring of enforcement of alcohol sales for underage drinkers to increase the awareness in the society, and among the retailers, which may contribute to better compliance and overall improvement of the public health in a country.
