# Supplementary material for: A mystery-shopping study to test enforcement of minimum legal purchasing age in Lithuania in 2022
Source: Eur J Public Health. 2023 Feb 25;33(2):317–22. doi: 10.1093/eurpub/ckad027 (PMC10066479; doi:10.1093/eurpub/ckad027)
Supplement: ckad027_Supplementary_Data [file ckad027_supplementary_data.docx]

Appendix 1. Figure 1 - Experimental study phases


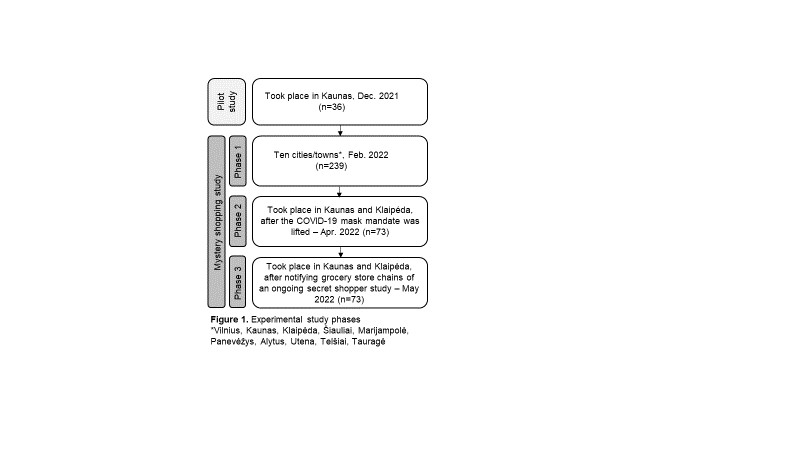


Appendix 2. Figure 2 – Mystery shopping action algorithm.


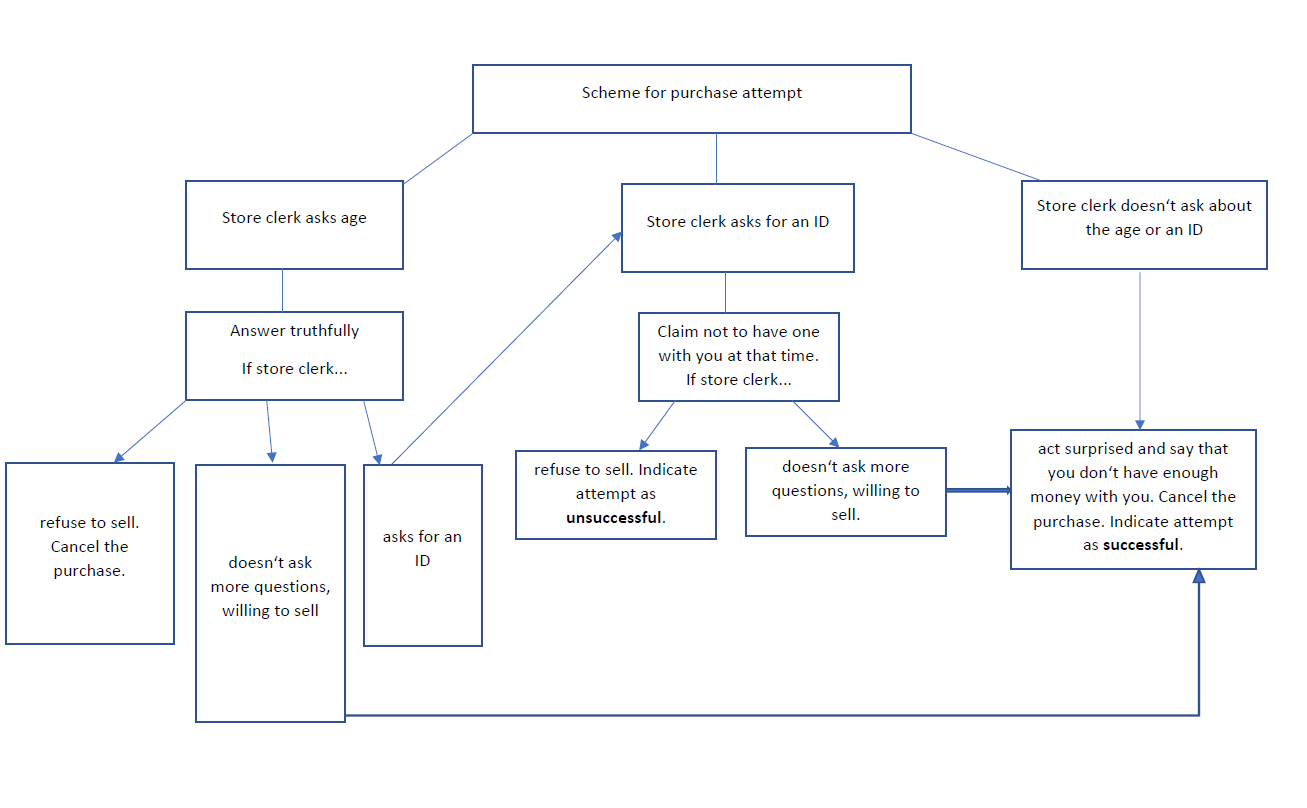


Appendix 3. Table 1 - Questionnaire

| Name of selling point |  |
| --- | --- |
| Location of selling point: |  |
| Day of visit: |  |
| Time of visit: |  |
| Gender of mystery shopper: | □ man □ woman |
| How many counters are in the selling point? |  |
| How many counters are open? |  |
| How many people were in front of you in line? |  |
| How many people were behind you in line? |  |
| Gender of seller: | □ man □ women |
| Estimated age of seller: |  |
| Did the seller ask for your ID? | □ yes □ no |
| Did the seller ask for your age | □ yes □ no |
| Could you buy alcohol? | □ yes □ no |
| Type of alcohol: | □ beer □ wine |
| Success / Fail (mark) | |
| Notes: | |

Appendix 4. Letter

| Researchers from the Health Research Institute of the Lithuanian University of Health Sciences are implementing an international research project "Evaluation of the impact of alcohol control policies on morbidity and mortality in Lithuania and other Baltic states". One of the parts of this project aims to get to know how alcohol control measures are implemented, and to achieve this goal, a mystery shop intervention on age verification is implemented. During this research, a buyer (a young person of legal age, who can purchase alcohol (20-24 years old)) tries to purchase alcoholic beverages to check whether a document that can confirm the person's age is required.  We inform you that in May 2022 one of the research phases is implemented and "mystery shoppers" will visit your chain stores. |
| --- |
